# Lactoferrin-Containing Immunocomplexes Drive the Conversion of Human Macrophages from M2- into M1-like Phenotype

**DOI:** 10.3389/fimmu.2018.00037

**Published:** 2018-01-23

**Authors:** Chen-Hui Gao, Hong-Liang Dong, Li Tai, Xiao-Ming Gao

**Affiliations:** ^1^Institute of Biology and Medical Sciences, Soochow University, Suzhou, China

**Keywords:** immune complex, lactoferrin, M2 macrophages, hyperactivity, rheumatoid arthritis

## Abstract

Macrophages are multifunctional cells that perform diverse roles in health and disease and considered the main source of inflammatory cytokines in affected joints of patients with rheumatoid arthritis (RA). M2 macrophages are well known as anti-inflammation and wound-healing cells; however, recent evidence suggests that they can also promote inflammation in RA, although the underlying mechanism remains to be clarified. Based upon our recent finding that lactoferrin (LTF)-containing IgG immunocomplex (LTF-IC), found elevated in RA sera, potent activators of human monocytes/macrophages, we herein demonstrate that LTF-IC was able to elicit immediate proinflammatory cytokine production by M2-polarized human macrophages through coligation with CD14/toll-like receptor (TLR) 4 and FcγRIIa (CD32a). The LTF-IC-treated M2 cells adopted surface maker expression profile similar to that of M1 phenotype and became functionally hyperactive to subsequent stimuli such as lipopolysaccharide, zymosan and IL-1β, which could provide a positive feedback signal to promote excessive inflammation in RA. They also acquired the ability to facilitate activation of Th17 cells that are known to play critical roles in RA pathology. We propose that IgG ICs containing TLR agonizing autoantigens are able to directly switch human macrophages from M2 into M1-like phenotype, thereby promoting excessive inflammation in autoimmune diseases such as RA.

## Introduction

Macrophages exhibit phenotypical and functional plasticity and can acquire a continuum of polarization states depending on the environmental cues (1–5). Classically activated macrophages (M1) are characterized by elevated expression of MHC class II, release of proinflammatory cytokines, generation of reactive oxygen species, and tumoricidal activity. Alternatively activated macrophages (M2), on the other hand, express high levels of anti-inflammatory cytokine IL-10, vascular endothelial growth factor, cyclooxygenase-2-derived PGE2 and possess tumor-promoting activity (6). In general, M1 macrophages predominate at the initial stages of inflammatory responses, whereas M2 macrophages drive the resolution of inflammation and tissue repair after injury and maintain tissue homeostasis (1, 2). It has been reported that transformation of tissue macrophages from M2 to M1 phenotype can occur *in vivo* which may hold the key to the break of self-tolerance and immune-pathogenic damage in autoimmune diseases (7–10).

Rheumatoid arthritis (RA) is a systemic autoimmune disorder characterized by chronic progressive joint inflammation that affects approximately 1% of the population worldwide (11–15). Production of autoantibodies is a hallmark of systemic autoimmune diseases such as RA and accumulating evidence suggests that immunocomplexes (ICs) between autoantibodies and self-antigens are pivotal pathogenic players *in vivo*, particularly through triggering Fc receptors (FcγRs) on infiltrating monocytes or tissue-resident macrophages. Monocytes/macrophages are the main source of proinflammatory cytokines (IL-1β, IL-6, and TNFα), which act both locally and systemically and are currently the main targets for RA immunotherapy (7–9). Both M1 and M2 macrophages could be identified in inflamed synovia of RA patients (9, 16), where the M1 cells were considered as the “bad guys,” while M2 the opposite. However, it has recently been shown that, after exposure to plate-coated IgG (representing insoluble IgG ICs deposited in local tissues) plus bacterial lipopolysaccharides (LPSs), a prototype Toll-like receptor (TLR)-4 agonist, M2 macrophages strongly produced inflammatory cytokines, indicating that costimulation through FcγRs and TLRs could drive M2-M1 macrophages conversion (10). Since some of the major autoantigens, such as lactoferrin (LTF) and citrilunated fibrinogen, in RA patients are also TLR agonists (17, 18), we propose that RA-related ICs might be able to drive the conversion of M2 macrophages into M1-like phenotype *via* synergistic signaling through TLRs and FcγRs.

Lactoferrin is an ~80 kDa multifunctional iron-binding glycoprotein of the transferrin family found in most mammalian exocrine secretions as well as secondary granules of neutrophils (19, 20). LTF-specific IgG autoantibodies are found in patients with various autoimmune diseases as RA, systemic lupus erythematosus (SLE), and antineutrophil cytoplasmic Ab-positive autoimmune vasculitis (21–23). We recently reported that LTF-containing ICs (LTF-ICs) are potent activators of human monocytes/macrophages (17). In the present study, LTF-IC was taken as a representative RA-related IC for investigation of their ability to drive the conversion of M2 into M1-like phenotype of human macrophages. Results from this study will have important implications for our understanding on the role of ICs in RA.

## Materials and Methods

### *In Vitro* Macrophage Differentiation

Peripheral blood mononuclear cells (PBMCs) were isolated from heparinized peripheral blood from healthy donors (HDs) by density gradient centrifugation at 500 *g* for 30 min on Ficoll lymphocyte separating solution (Dakewe Biotech) at room temperature. All donors gave written informed consent to participate in the study. CD14+ blood monocytes were purified from PBMCs by magnetic cell sorting using CD14 microbeads (Miltenyi Biotec, Germany) and used for generation of M1 and M2 macrophages following the protocol of Vogelpoel et al. (10). Monocytes were cultured for 6 days in RPMI 1640 (Hyclone) containing 10% fetal bovine serum (FBS, Biological Industries) supplemented with 20 ng/ml recombinant human M-CSF (Peprotech) for M2, or 500 U/ml recombinant human GM-CSF (Peprotech) for M1 macrophages. For M2, at day 3, half of the medium was replaced by new medium containing cytokines. At day 7, the medium was totally replaced in the presence of 20 ng/ml recombinant human IL-4 (Peprotech), respectively. Since the new guideline for *in vitro* polarization of M1 and M2 macrophages (1) was not followed in the present study, the differentiated M1 and M2 cells herein prepared are specified (GM-CSF)-M1 and (M-CSF)-M2 macrophages, respectively.

### Preparation of LTF-ICs

Anti-huLTF antibodies from RA patients (LTF-Abs) and LTF-ICs were prepared as previously described (17). Briefly, LTF-Abs were sequentially purified by affinity chromatography on LTF-S4B (prepared in our laboratory) and Protein A-S4B columns (Pierce) from six pooled plasma samples shown by ELISA to contain high levels of anti-LTF antibodies. The eluted IgG fractions were concentrated by centrifugation with buffer exchange to phosphate-buffered saline (PBS) (Amicon Ultra, Millipore) and were depleted of endotoxin by filtration through a polymyxin B column (Detoxigel). IgG concentrations were determined by optical density at 280 nm; IgG was aliquoted, and stored at −80°C. Preparation and characterization of a mouse mAbs against huLTF (M860), bovine serum albumin (BSA) (J1) or chicken ovalbumin (M562) in this laboratory have also been documented (24). For preparation of LTF-IC, human LTF (2 µg/ml, Sigma-Aldrich) and M860 (2 µg/ml, mAbs of LTF, purified with protein G antibody affinity chromatography, GE Healthcare Life Sciences) or LTF-Abs were mixed in a sterile tube with gentle rotation at 37°C for an hour. IC between LTF and M860 were separated from the uncoupled Ab and antigen using Sephadex Superfine G-75 column. The elutions of IC were pooled, desalted and concentrated. Endotoxin was removed by polymyxin B coupled beads repeatedly and the level of endotoxin in IC was below 1 EU/mg which was detected by Chromogenic LAL Endotoxin Assay Kit (Genscript). ICs between BSA (Sigma-Aldrich) and J1 were used as control and prepared similarly.

### Flow Cytometry

For phenotyping, cells were collected, washed with PBS, and the pellets were incubated for 30 min at 4°C with 50 µl APC-conjugated mouse anti-human CD14, CD163, CD16, or CD32, or PE-conjugated mouse anti-human CD86 or CD206, or FITC-conjugated mouse anti-human CD64, or APC-, PE-, FITC-conjugated isotype control Abs (Biolegend). After washes, the cells were subjected to analysis by flow cytometry Attune NxT (Life Technology).

### Stimulations and ELISAs

*In vitro* differentiated macrophages were harvested by gentle pipetting and stimulated (3–5 × 10^4^ cells/well) with 30 µg/ml LTF, M860 (LTF-Abs), M860-IC (LTF-IC), or 100 ng/ml LPS (from *Escherichia coli* 0111:B4; Sigma-Aldrich) in 96-well plates (Nunc) for 18 h in a 5% CO_2_ incubator at 37°C, then the supernatants were collected and stored at 4°C, until analysis by ELISA.

For PAMPs and inflammatory cytokine restimulation assays, LTF-IC-pretreated macrophages (5 × 10^4^ cells/well) were restimulated with different stimuli, including 10 ng/ml LPS, 1 µg/ml zymosan and 1 µg/ml curdlan, or with cytokines including 10 ng/ml IL-1β, 1,000 U/ml IFN-α/β, 2,000 U/ml IFN-γ, 100 ng/ml IL-6, 5 ng/ml IL-12, and the cell supernatants were collected. Cytokine levels were determined by TNFα ELISA kit.

The levels of TNFα, IL-1β, IL-6, IL-10, IL-17, IL-23, and IFN-γ in human macrophage culture supernatants were measured by using ELISA kits (from eBioscience) according to the manufacturer’s instructions. Standard curves were established using human recombinant cytokines provided in the kits.

### T Cell Activation

Memory T cells were isolated from PBMCs of HDs using flow sorting (Arial III, BD Biosicences) stained with anti-CD45RO-PE (Biolegend) and anti-CD4-APC (MiltenyiBiotec). For *in vitro* activation of T cells, 2 × 10^5^ (M-CSF)-M2 macrophages were stimulated with 30 µg/ml LTF, M860, M860-IC or 100 ng/ml LPS, and then cocultured with 2 × 10^5^ allogeneic memory CD45RO^+^CD4^+^ T cells in presence of 2 µg/ml anti-IL-4 (R&D) and 10 µg/ml anti-IFN-γ (BD Pharmingen), costimulated with 100 ng/ml anti-CD3 antibodies (eBioscience) coated in the plates. Every 2 days, half of the medium was replaced by RPMI 1640 (Hyclone) containing 10% FBS and 20 U/ml recombinant human IL-2 (Peprotech). After 4 days, cells were transferred to 96-well flat-bottomed culture plates (Nunc).

For intracellular cytokine staining, T cells were restimulated by cell stimulation cocktail (including PMA, ionomycin, brefeldin A, and monensin, eBioscience) for 6 h. Cells were harvested and washed, fixed with fixation buffer (Biolegend) for 20 min at room temperature, washed again, permeabilized with Intracellular staining perm wash buffer (Biolegend) for 30 min. Cells were incubated with anti-IL-17-FITC (1:50; MiltenyiBiotec) and anti-IFN-γ-APC (1:50; MiltenyiBiotec) for 60 min at room temperature and analyzed by flow cytometry (Canto II, BD Biosicences). For cytokine analysis, resting T cells were restimulated with 1 µg/ml anti-CD3 and 1 µg/ml anti-CD28 (eBioscience). Supernatants were harvested after 24 h and stored at 4°C until analysis by ELISA.

### Quantitative RT-PCR

For mRNA-level analysis, cells were lysed at the indicated time points, after which mRNA extraction was performed using Omega RNA Isolation Kit and cDNA synthesis using First Strand cDNA Synthesis Kit (Takara). Quantitative RT-PCR (StepOnePlus real-time PCR system; Applied Biosystems, Life Technology), was performed using SYBR green (Takara) and primer pairs as listed in Table 1. The mRNA levels were normalized to housekeeping gene expression [2^Ct(housekeeping) − Ct(target)^], and folds were calculated compared with an unstimulated control sample.

**Table 1:** Primers used in this study.

Target mRNA	Forward primer (5′–3′)	Reverse primer (5′–3′)
**Human**		
GAPDH	GAAGGTGAAGGTCGGAGTC	GAAGATGGTGATGGGATTT
TNFA	GGCTCCAGGCGGTGCTTG	CAGATAGATGGGCTCATACCA
IL1B	TTTGAGTCTGCCCAGTTCCC	TCAGTTATATCCTGGCCGCC
*IL6*	TGACAAACAAATTCGGTACATCCT	AGTGCCTCTTTGCTGCTTTCAC
*IL23A*	GTGGGACACATGGATCTAAGAGAAG	TTTGCAAGCAGAACTGACTGTTG
*IL12A*	AGTGCCGGCTCAGCATGTGT	GTGGCCACGGGGAGGTTTCT
*IL12B*	ACGTTTCACCTGCTGGTGGCT	CTCCGCACGTCACCCCTTGG
IL10	ATGCTTCGAGATCTCCGAGA	AAATCGATGACAGCGCCGTA
FOLR2	CCTGCAGGGACAGAAAGACA	CCAGGGACTGCATTGGTCAT
*SLC40A1*	TATTCATGCCTGGAAGCCCC	TTCTAGCAGCAATGACGCCT
*HMOX1*	CTGCGTTCCTGCTCAACATC	ATCTTGCACTTTGTTGCTGGC
ALDH1A2	TGGCAGAATCCTTTTTGGGAGA	TCCATGGTTGCAAGAACTGC
INHBA	AAGTCGGGGAGAACGGGTAT	TCTTCCTGGCTGTTCCTGACT
CLEC5A	CCTTTGCCAAGAACCCCACT	GGGCAGACTGTTCCATAGCTC

### Antibody and Inhibitor Blockade

Blocking antibodies were preincubated with (M-CSF)-M2 macrophages for 2 h in a 5% CO_2_ incubator at 37°C, after which stimuli and culture medium were added resulting in a final antibody concentration of 5 µg/ml anti-CD16/32/64 and 2 µg/ml anti-CD14. Syk or TLR4 was inhibited by incubating M2 macrophages with titration of R406, Belnacasan VX-765 (both from Selleckchem) or CLI-095 (Invitrogen) for 2 h at 37°C before stimulation.

### Statistical Analysis

All experiments were repeated at least three times and the results are expressed as mean ± SD. Comparison of the data was performed using the Student’s *t*-test. Significance was defined as a *P* value of <0.05%.

### Ethics Statement

This study was approved by the Ethics Committees of Soochow University Medical School, Suzhou, China. Written informed consent was obtained from all participants prior to inclusion in the study.

## Results

### Proinflammatory Cytokine Production by Human (M-CSF)-M2 Cells following LTF-IC Stimulation

Human (GM-CSF)-M1 and (M-CSF)-M2 macrophages were generated *in vitro* by treating freshly purified human CD14^+^monocytes (Figure S1A in Supplementary Material) for 7 days with GM-CSF or M-CSF plus IL-4, respectively. The resultant cells displayed expected surface marker expression profiles of M1 (CD86 and CD64^high^) or M2 (CD14, CD163, and CD16^high^) phenotypes (Figure S1B in Supplementary Material). Q-PCR results confirmed mRNA transcription for the genes of ALDH1A2, CLEC5A, and INHBA in the (GM-CSF)-M1 and SLC40A1, FOLR2, and HMOX1in the (M-CSF)-M2 cells (Figure S1C in Supplementary Material), which is consistent with the characteristic gene expression profile of M1 and M2 macrophages. As expected, (GM-CSF)-M1 cells readily produced inflammatory cytokine TNFα, while (M-CSF)-M2 produced anti-inflammatory cytokine IL-10 in response to subsequent LPS stimulation (Figure S1D in Supplementary Material).

**Figure 1:**
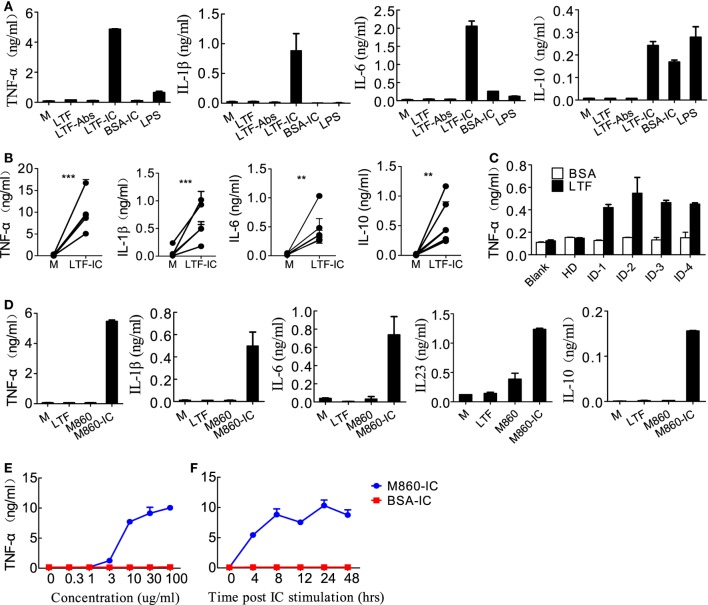
Lactoferrin-containing IgG immunocomplex (LTF-IC) induced proinflammatory cytokines production by human (M-CSF)-M2 macrophages. **(A)** Freshly differentiated (M-CSF)-M2 macrophages (5 × 10^4^ cells/well) were stimulated with 30 µg/ml human LTF (hLTF), LTF-Abs, LTF-IC, bovine serum albumin (BSA)-IC, or 100 ng/ml lipopolysaccharide(LPS) for 18 h, followed by quantitation of TNFα, IL-1β, IL-6 and IL-10 in culture supernatant using ELISAs. **(B)** (M-CSF)-M2 macrophages differentiated from different donors (*n* = 5) were treated with, or without (M), LTF-IC for 18 h, followed by cytokine quantitation using ELISAs. Each pair of dots represents one donor. **P < 0.01. ***P < 0.001. **(C)** Serum samples (diluted 1:100 in PBS) from healthy donors (HDs) or four rheumatoid arthritis patients with high titer LTF-specific autoantibodies were dispensed into 96-well cell culture plates precoated with, or without, hLTF and incubated for 2 h. (M-CSF)-M2 macrophages were then added to the wells and incubated for 18 h, followed by quantitation of TNFα in culture supernatant by ELISAs. **(D)** (M-CSF)-M2 macrophages were cocultured with 30 µg/ml hLTF, M860 or M860-IC for 18 h followed by quantitation of TNFα, IL-1β, IL-6, IL-23, and IL-10 in culture supernatant using ELISAs. **(E)** (M-CSF)-M2 macrophages were stimulated with increasing concentrations of M860-IC or BSA-IC (0–100 µg/ml) for 18 h followed by ELISA quantitation of TNFα in culture supernatant. **(F)** Time course of (M-CSF)-M2 macrophage response to stimulation of 30 µg/ml M860-IC or BSA-IC in terms of TNFα production. The data are expressed as mean ± SD performed in parallel and representative of at least three experiments with different donors.

Based on our recent finding that LTF-IC was able to coligatie TLR4 and FcγRIIa on human monocytes/macrophages and induce a strong TNFα response (17), we wondered whether LTF-IC could also elicit inflammatory cytokine production by human (M-CSF)-M2 cells *in vitro*. In the experiment shown in Figure 1, (M-CSF)-M2 cells were stimulated with a mixture (1:1) of human LTF (hLTF) and LTF-specific IgG autoantibodies (affinity purified from RA sera) for 18 h followed by quantitation of TNFα, IL-1β, IL-6, and IL-10 in the culture supernatant. Clearly LTF-IC, but not anti-LTF IgG or hLTF alone, or huLTF plus control hIgG, was able to elicit production of TNFα, IL-1β, IL-6, and IL-10 by (M-CSF)-M2 cells (Figure 1A), while LTF-IC treatment had minimal effect on (GM-CSF)-M1 macrophages (Figure S2A in Supplementary Material). There was considerable individual variation in TNFα response of (M-CSF)-M2 cells generated from PBMCs of different donors, but the trend of all individual samples remained consistent (Figure 1B). Based on LTF-specific IgG Ab screening results of our earlier study on RA sera (17), serum samples from four patients with high titer anti-LTF autoantibodies were added to wells precoated with hLTF, the plate-bound ICs thus formed exhibited capability to induce TNFα production by (M-CSF)-M2 cells (Figure 1C).

**Figure 2:**
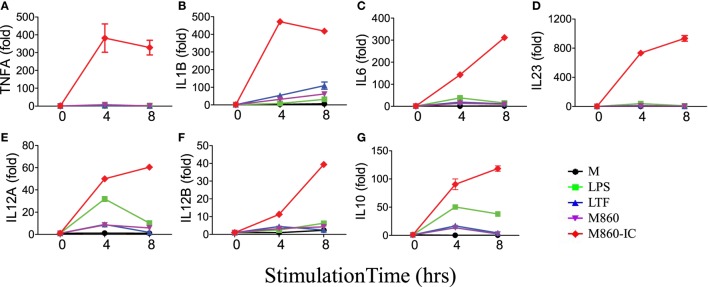
M860-IC enhances proinflammatory cytokines transcription in M2. (M-CSF)-M2 macrophages were stimulated with, or without (M), 30 µg/ml human lactoferrin, M860, M860-IC, or 100 ng/ml lipopolysaccharide (LPS) for 4 or 6 h and then analyzed for mRNA expression of **(A–G)** indicated genes (normalized to GAPDH expression, fold increase compared with unstimulated controls) by quantitative RT-PCR at indicated time points. The results are representative of three experiments with different donors.

Like autoantibody-containing LTF-ICs, complex between hLTF and mouse anti-hLTF mAb M860 (M860-IC) is also capable of eliciting proinflammatory cytokine, but not IL-10, production by human monocytes (17), which can be explained by the fact that mouse IgG1 binds human FcγRs with relatively high affinity (26, 27). In the present study, M860-IC strongly triggered (M-CSF)-M2 secrete of TNFα, IL-1β, IL-6, IL-10, and IL-23 (Figure 1D). In subsequent experiments M860 was employed as a replacement of human autoantibodies against hLTF. Dose curve of M860-IC-induced TNFα production by (M-CSF)-M2 cells, shown in Figure 1E, indicates that minimal concentration for LTF-IC to activate (M-CSF)-M2 cells was approximately 3 µg/ml. TNFα secretion by M860-IC-treated (M-CSF)-M2 cells was readily detectable after 4 h stimulation (Figure 1F). The above results are further confirmed by Q-PCR data showing strongly increased mRNA transcription of *TNFA, IL1B, IL6, IL12A, IL12B*, and *IL23A* genes in (M-CSF)-M2 macrophages after 4–6 h exposure to LTF-IC (Figure 2).

### LTF-IC-Mediated Transformation of M2 into M1-Like Cells *In Vitro*

**Figure 3:**
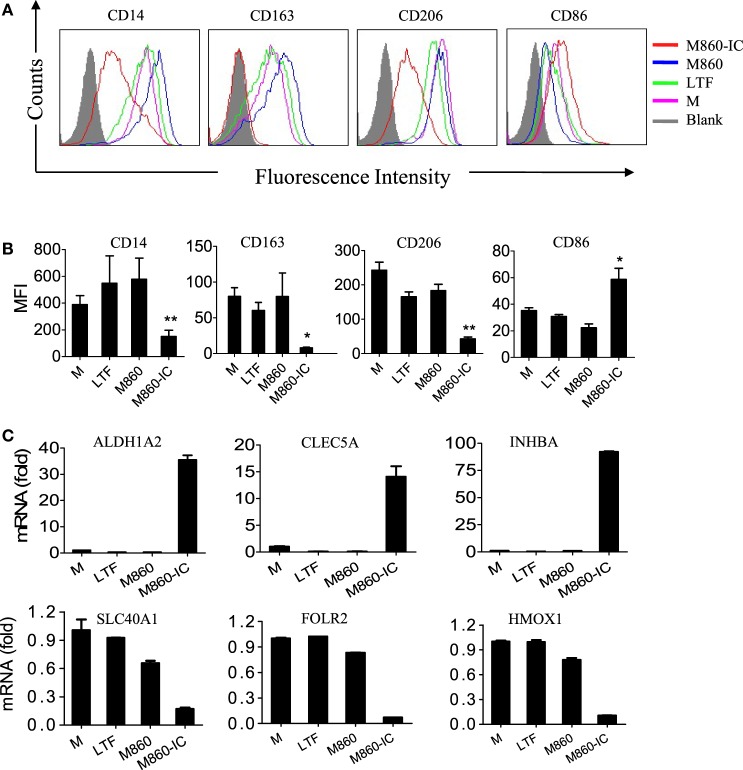
Effects ofM860-ICon human macrophage polarization. **(A)** (M-CSF)-M2 macrophages were stimulated with, or without (M), 30 µg/ml lactoferrin (LTF), M860, M860-IC for 18 h, then the collected cells were stained with APC-conjugated anti-human CD14, APC-conjugated anti-human CD163, PE-conjugated anti-human CD206, or CD86 for flow cytometric analysis. Mean fluorescence intensity were analyzed in **(B)** of three independent experiments **(B)**. **(C)** (M-CSF)-M2 macrophages were stimulated with 10 µg/ml LTF, M860, and M860-IC for 18 h, then the collected cells were analyzed for mRNA expression of M1-specific markers including SLC40A1, FOLR2, and HMOX1, and M2-specific markers including ALDH1A2, CLEC5A, and INHBA (normalized to GAPDH expression, fold increase compared with unstimulated controls) by quantitative RT-PCR. The results are representative of three experiments from different donors.

**Figure 4:**
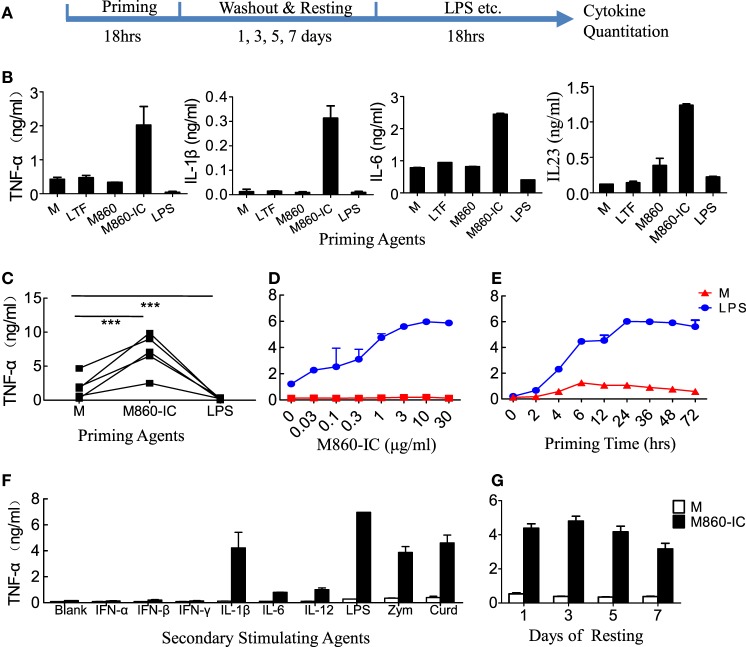
Hyper-responsiveness of M860-IC-treated M2 cells. **(A)** Diagram showing the course of the experiment examining hyper-responsiveness of the lactoferrin (LTF)-containing IgG immunocomplex (LTF-IC)-primed M2 cells. **(B,C)** (M-CSF)-M2 macrophages were primed for 18 h with, or without (M), 30 µg/ml human LTF (hLTF), M860, M860-IC, or 100 ng/ml lipopolysaccharide(LPS), followed by washout and resting and then a 24 h restimulation with suboptimal concentration (100 ng/ml) LPS. Cytokines in culture supernatants were quantitated using ELISAs. Data from different donors (*n* = 5) are further compared in **(C)**, each pair of dots represents one donor. ***P < 0.001. **(D)** Dose curve and **(E)** time course of M860-IC priming of (M-CSF)-M2 macrophages. The results are expressed as TNFα secretion by the LTF-IC-primed M2 cells in response to a 18 h stimulation with, or without (M), 100 ng/ml LPS. **(F)** (M-CSF)-M2 macrophages pretreated with, or without (M), M860-IC were restimulated with a panel of stimuli, including LPS (100 ng/ml), zymosan (1 µg/ml), curdlan (1 µg/ml), IL-1β (10 ng/ml), IFN-α/β (1,000 U/ml), IFN-γ (2,000 U/ml), IL-6 (100 ng/ml), or IL-12 (5 ng/ml), for 24 h. **(G)** (M-CSF)-M2 macrophages pretreated with, or without (M), M860-IC were allowed to rest 1, 3, 5 or 7 days in fresh medium, and then challenged with LPS (100 ng/ml) for TNFα production. The results, expressed as mean ± SD, are representative of three experiments with different donors.

Lactoferrin IC treatment of (M-CSF)-M2 macrophages resulted in not only inflammatory cytokine secretion but also a switch from M2-specific (CD14, CD163, CD206, SLC40A1, FOLR2, and HMOX1) to M1-specific (CD86, ALDH1A2, CLEC5A, and INHBA) marker expression as evidenced by FACS and Q-PCR analysis (Figure 3). In addition to the phenotypical changes, LTF-IC-primed (M-CSF)-M2 macrophages acquired a significantly enhanced functional state in that they remained hyperactive to LPS stimulation for several days. As shown in Figure 4, LTF-IC-primed (M-CSF)-M2 macrophages (12 h stimulation followed by washes and a resting period of 24 h) vigorously responded to suboptimal concentration LPS by producing large amounts of TNFα, IL-1β, IL-6, and IL-23, albeit the ability to make IL-10 was not significantly affected (data not shown). By contrast, LPS-primed (M-CSF)-M2 became unresponsive to subsequent LPS stimulation. Although there was considerable individual variation in LPS responsiveness (TNFα production) by LTF-IC-primed (M-CSF)-M2 cells from different donors, the trend of all donors remained consistent (Figure 4C). Dose curve and time course studies (Figures 4D,E) indicate that a 4 h treatment with LTF-IC at a dose of 0.3 µg/ml was enough to make (M-CSF)-M2 macrophages significantly more responsive (LPS-induced TNFα secretion) than unprimed controls. More importantly, LTF-IC-primed (M-CSF)-M2 macrophages were high responders to not only LPS but also β-glucans (zymosan and curdlan) and IL-1β (Figure 4F). Though not as impressive, their responsiveness to IL-6 and IL-12, but not IFNs, was also significantly increased. Finally, the hyperactivity of LTF-IC-primed (M-CSF)-M2 macrophages lasted for up to 7 days *in vitro* (Figure 4G). It should be noted that LPS-induced TNFα production in (GM-CSF)-M1 macrophages was significantly down-regulated by LTF-IC (Figure S2B in Supplementary Material).

### LTF-IC Steers M2-M1 Conversion through CD14/TLR4 and CD32 Cross-linking

**Figure 5:**
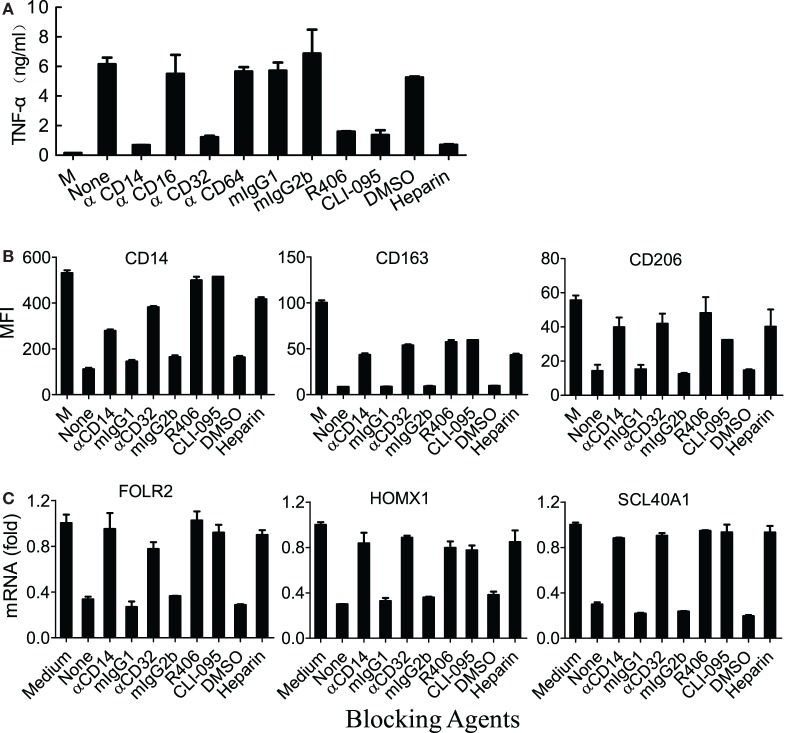
M860-IC induced phenotype switching is dependent on CD14 and CD32. **(A)** (M-CSF)-M2 macrophages were exposed to M860-IC (30 µg/ml) for 18 h in the presence of heparin or R406 (0, 1, 3 µM), mAbs against human CD14, CD16, CD32, CD64, or CLI-095 (5 µM). Isotype-matched irrelevant Abs as well as untreated (M-CSF)-M2 cells (M) were included as controls. TNFα in the culture supernatant was quantitated using ELISAs. **(B)** (M-CSF)-M2 macrophages were exposed to M860-IC (30 µg/ml) for 18 h in the presence, or absence (None), of anti-CD14 (2 µg/ml), anti-CD32 (5 µg/ml), R406 (3 µM), CLI-095 (5 µM), or heparin (10 µM). Isotype-matched mAbs and DMSO were included as controls. The cells were then stained with fluorescence-labeled mAbs against human CD14, CD163 or CD206 for flow cytometric analysis. The results are shown as mean fluorescence intensity (MFI). **(C)** The above cells were also harvested and analyzed for mRNA expression of M2-specific markers including ALDH1A2, CLEC5A, INHBA (normalized to GAPDH expression, fold increase compared with un-stimulated control) by quantitative RT-PCR. The results are expressed as mean ± SD performed in parallel and representative of three experiments with different donors.

CD14/TLR4 is known to be the main surface receptor in monocytes for interaction with LTF, and LTF-IC activation of human monocytes was susceptible to suppression by heparin (blocker of LTF and receptor binding), blocking Abs against CD14 or CD32 as well as TLR4-specific inhibiting agents (17). Similarly, TNFα production by LTF-IC-treated (M-CSF)-M2 macrophages was significantly blocked by heparin, CLI095 (TLR4-specific chemical inhibitor), and mAbs against human CD14 or CD32, but not CD16 or CD64 (Figure 5A). Consistently, R406, a chemical inhibitor of CD32a signal transduction molecule Syk, dose dependently blocked LTF-IC-induced (M-CSF)-M2 activation (Figure 5A). Furthermore, LTF-IC-mediated decrease in surface expression of CD14, CD163, and CD206 molecules (Figure 5B) and suppression of M2 signature genes (SLC40A1, FOLR2, and HMOX1) (Figure 5C) in (M-CSF)-M2 cells were significantly reversed by heparin, CLI095, R406 or blocking mAbs against human CD14 or CD32. Taken together, both the CD14-TLR4 pathway and CD32-Syk axis play pivotal roles in LTF-IC-mediated (M-CSF)-M2 activation and subsequent conversion to an M1-like phenotype. Next we asked whether costimulation by unconjugated TLR agonist(s) and deposited IgG could also achieve similar M2 to M1 switch. As summarized in Table 2, combination of plate-coated IgG and soluble LTF was as effective as LTF-IC in eliciting IL-1β, IL-6, and TNFα secretion by (M-CSF)-M2 cells *in vitro*.

**Table 2:** Cross-talk between FcγR and LTF-R in human M2 macrophages.^a^

Cytokine quantitation^b^	Stimulators			
	Medium	cIgG	hLTF	cIgG + hLTF
IL-1β (pg/ml)	26.25 ± 3.64	4.95 ± 2.75	29.47 ± 0.91	558.73 ± 115.30
IL-6(pg/ml)	23.27 ± 12.30	29.60 ± 1.12	39.90 ± 11.21	636.88 ± 45.66
TNFα (pg/ml)	67.65 ± 7.55	844.0 ± 76.77	62.31 ± 3.78	5,180.38 ± 167.87

*Data are shown as mean ± SD and representative of three experiments using blood samples from different donors*.

*^a^Freshly differentiated human (M-CSF)-M2 macrophages were stimulated with cIgG (10 µg/ml), or hLTF(30 µg/ml), or cIgG + hLTF for 18 h followed by quantitation of cytokines in the supernatant*.

*^b^Cytokine levels were determined by TNFα, IL-1β, and IL-6 ELISA kit. Data are shown as mean ± SD and representative of 3 experiments using blood samples from different donors*.

### LTF-IC-Primed M2 Macrophages Promote Th17 Activation

**Figure 6:**
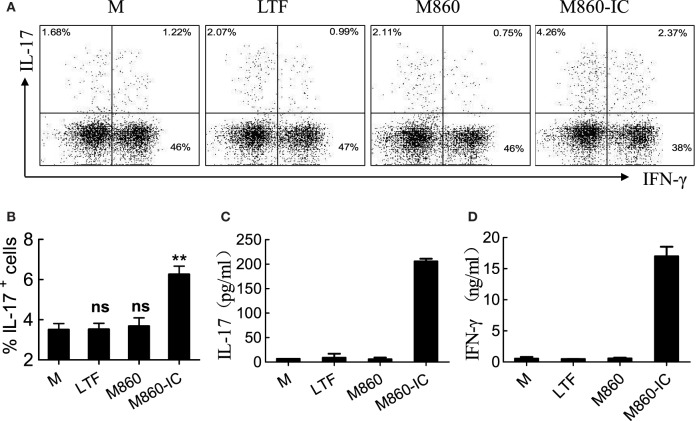
(M-CSF)-M2 treated with lactoferrin (LTF)-containing IgG immunocomplex (LTF-IC) promotes Th17 activation. Freshly isolated peripheral blood CD4^+^CD45RO^+^ T cells were cultured together with allogeneic (M-CSF)-M2 macrophages that had been pretreated with, or without (M), 30 µg/ml human LTF (Hltf), M860, or M860-IC in tissue culture plates precoated with 100 ng/ml anti-CD3 antibodies for 4 days, followed by restimulation with cell stimulation cocktail. IL-17 **(A)** and IFN-γ **(B)** in the supernatants were determined by ELISA. The cells were intracellularly double-stained using FITC-conjugated anti-IL-17 and APC-conjugated anti-IFN-γ for FACS analysis gating on CD4^+^ T cells **(C)**. Statistics comparing percent IL-17-positive cells amongst CD4 T lymphocytes in different groups of these cultures are shown **(D)**. **P < 0.01. ns, not significant. Data (mean ± SD) are representative of three independent experiments with three biological replicates.

Macrophages are regarded as antigen-presenting cells capable of inducing CD4^+^ T helper (Th) cell activation and polarization through cytokines (e.g., IL-1β, IL-6, and TNFα) they produce (28–30). It has also been reported that IgG-opsonized bacteria were able to promote human Th17 response *via* synergy between TLRs (TLR2, 4, 5) and FcγRIIa in dendritic cells (31). It is therefore reasonable to question whether LTF-IC-primed M2 macrophages could facilitate Th17 cell activation and/or polarization in a similar fashion. In the experiment shown in Figure 6, CD4^+^ T cells, freshly purified from PBMC of HDs, were cultured together with LTF-IC-stimulated (M-CSF)-M2 macrophages for 96 h, followed by quantitation of IL-17 and IFNγ in the culture supernatant and intracellular staining for the same cytokines in CD4^+^ cells. Percentage of IL-17^+^Th cells in the LTF-IC-primed group was significantly higher than that of the controls as evidenced by intracellular staining (Figures 6A,B). Apparently CD4^+^ T cells strongly responded to LTF-IC-primed (M-CSF)-M2 cells, but not the controls, by producing large amounts of IL-17 and IFN-γ (Figures 6C,D).

## Discussion

An important finding of this study is that LTF-IC is able to not only elicit immediate proinflammatory cytokine production by human (M-CSF)-M2 macrophages but also drive their transformation into M1-like phenotype with sustainable hyperactivity. Interestingly, LTF-IC-primed (M-CSF)-M2 cells are also able to facilitate the activation of memory Th17 cells, a cell type highly activated both systemically and locally in inflamed synovium of RA patients (28). Note that LTC-ICs-treated (M-CSF)-M2 was unable to drive naive CD4 Th into IL-17-producing cells in similar experiments (data not shown). Our results provide additional clues for the pathological roles of ICs between autoantibodies and biologically active autoantigens. Given that LTF concentration can be elevated significantly in circulation or synovial fluid of RA patients, the concentration range of LTF-ICs (10–30 µg/ml) used in this study is pathophysiologically relevant. We propose that LTF-ICs can be considered as novel proinflammatory mediators contributing to the pathogenesis of autoimmune diseases such as RA by steering macrophage polarization toward proinflammatory M1-like phenotype.

Some of the TLR agonist-containing IgG ICs from patients with systemic autoimmune disease such as SLE and RA possess potent stimulatory activities on myeloid cells, mostly through synergistic signaling of FcγRIIa and TLRs (10, 17, 18). In the “dual signal activation model,” simultaneous ligation of FcγRIIa (*Signal 1*) and TLR (*Signal 2*) results in immediate production of proinflammatory cytokines. For instance, complexes between RA-related ACPA (anticitrilunated protein autoantibodies) and citrullinated fibrinogen or vimentin, could induce macrophage secretion of proinflammatory cytokines through FcγRIIa and TLR4 engagement (18). DNA-ICs found in SLE patients trigger activation cascade through cooperation of CD32 and TLR9 in monocytes/macrophages (32). It is reasonable to suggest that all such ICs between autoantibodies and biologically active autoantigens might also be able to endorse M2-M1 polarization and act in a concerted manner to play pivotal roles in initiating overt inflammatory tissue damage in disease conditions (8, 32). Dominguez-Soto and colleagues recently reported that IVIG, a preparation of polyclonal and poly-specific Igs derived from the plasma of thousands of HDs, impaired the effect and function of M1 macrophages, but on the other hand caused a M2-to-M1 polarization switch (33). In their study, however, very high concentration (10 mg/ml) of IgG was used. It is quite unlikely that a significant amount of TLR agonists-containing ICs were present in IVIG preparations derived from HDs rather than patients with autoimmune disorders.

It is long been accepted that synovial inflammation, and the production of proinflammatory and destructive mediators from activated M1 macrophage, are of importance for the symptoms and progression of RA, while M2 macrophages mediate anti-inflammatory effects by producing anti-inflammatory cytokines such as IL-10 and TGF-β (4, 7). Vogelpoel et al. recently reported that costimulation of macrophages through FcγRIIa (cIgG) and TLR4 (LPS) elicited proinflammatory cytokine production by human M2 macrophages *in vitro* (10). Our results provide additional clues for the pathological roles of M2 macrophages during RA process.

It has been demonstrated by previous investigators that human monocytes/macrophages can be trained, by exposure to *C. albicans* or β-glucans, to exhibit enhanced proinflammatory responsiveness and glycolysis (34–36). This “trained immunity” of monocytes/macrophages is mediated through Dectin-1/Raf-1 signaling pathway (34), which is different from that (FcγRIIa/Syk) triggered by LTF-IC. While functional polarization of macrophages induced by TLR agonist autoantigen-containing ICs are potentially pathological players in autoimmune disorders, β-glucan-trained macrophages displayed stronger ability in phagocytosis, indicative of more active roles in immunological defense and scavenging.

Implications for characterization of macromolecules capable of driving M2-M1 conversion go beyond the field of autoimmune diseases and infection immunity. Macrophages are the major tumor-infiltrating leukocytes and play a critical role in cancer-related inflammation (1, 2, 6), and depending on their polarization status, tumor-associated macrophages (TAMs) can either promote antitumor immune responses or contribute to tumor progression (6, 37, 38). The M2/M1 phenotype switch of TAM is especially relevant in tumor initiation, progression, and dissemination (37, 39). Currently, strategies to target M2 include depletion or blocking of recruitment (40, 41) and decreasing M2-like TAM *via* reeducation (42, 25). These approaches employ chemotherapeutic drugs, Abs, or small-molecule inhibitors, which may cause unwanted adverse effects. In this aspect, targeting TAM with TLR agonist-containing ICs (e.g., LTF-IC) might provide a new potential therapy for cancers.

## Author Contributions

H-LD and X-MG designed the research. C-HG, H-LD, and LT carried out the experiment. H-LD analyzed the data. H-LD and X-MG prepared the manuscript. All authors discussed the results and commented on the manuscript.

## Conflict of Interest Statement

The authors declare that the research was conducted in the absence of any commercial or financial relationships that could be construed as a potential conflict of interest.
