# Supplementary material for: Lactoferrin-Containing Immunocomplexes Drive the Conversion of Human Macrophages from M2- into M1-like Phenotype
Source: Front Immunol. 2018 Jan 23;9:37. doi: 10.3389/fimmu.2018.00037 (PMC5787126; doi:10.3389/fimmu.2018.00037)
Supplement: Supplementary file 1 [file Data_Sheet_1.docx]

Supplementary Material

**Lactoferrin-Containing Immunocomplexes Drive the Conversion of Human Macrophages from M2 into M1-like Phenotype**

**Chenhui Gao*, Hongliang Dong*, Li Tai*, and Xiaoming Gao**

*** Correspondence:** Corresponding author: Dr. HL Dong, [hldong@suda.edu.cn](mailto:hldong@suda.edu.cn); or Dr. XM Gao, [xmgao@suda.edu.cn](mailto:xmgao@suda.edu.cn);

# Supplementary Data

**1.1 In vitro macrophage differentiation.**

Peripheral blood mononuclear cells (PBMCs) were isolated from heparinized peripheral blood from HD by density gradient centrifugation at 500g for 30 min on Ficoll lymphocyte separating solution (Dakewe Biotech) at room temperature. The PBMCs were collected and washed twice with PBS. All donors gave written informed consent to participate in the study. Monocytes were purified from PBMCs by magnetic cell sorting using CD14 microbeads (MiltenyiBiotec, Germany). Macrophages were generated by culturing freshly separated monocytes for 6 days in RPMI 1640 (Hyclone) containing 10% fetal bovine serum (FBS, Biological industries) supplemented with 20 ng/ml recombinant human M-CSF (Peprotech) for (M-CSF)-M2 macrophages, or 500 U/ml recombinant human GM-CSF (Peprotech) for (GM-CSF)-M1 macrophages. For (M-CSF)-M2, at day 3, half of the medium was replaced by new medium containing cytokines. At day 7, the medium was totally replaced in the presence of 20 ng/ml recombinant human IL-4 (Peprotech), respectively.

**1.2** (**GM-CSF)-M1 stimulation.**

(GM-CSF)-M1 cells derived from PBMC of different donors were harvested and stimulated with or without M860-IC (30 μg/ml) for 18 hours in a 5% CO_2_ incubator at 37˚C, then the supernatants were collected and stored at 4°C, until analysis by ELISA. For LPS restimulation, (GM-CSF)-M1 macrophages treated by LTF-IC for 12 hours were re-stimulated with 10 ng/ml LPS, and the cell supernatants were collected. Cytokine levels were determined by TNFα ELISA kit.

# Supplementary Figures and Tables

# 2.1 Supplementary Figures


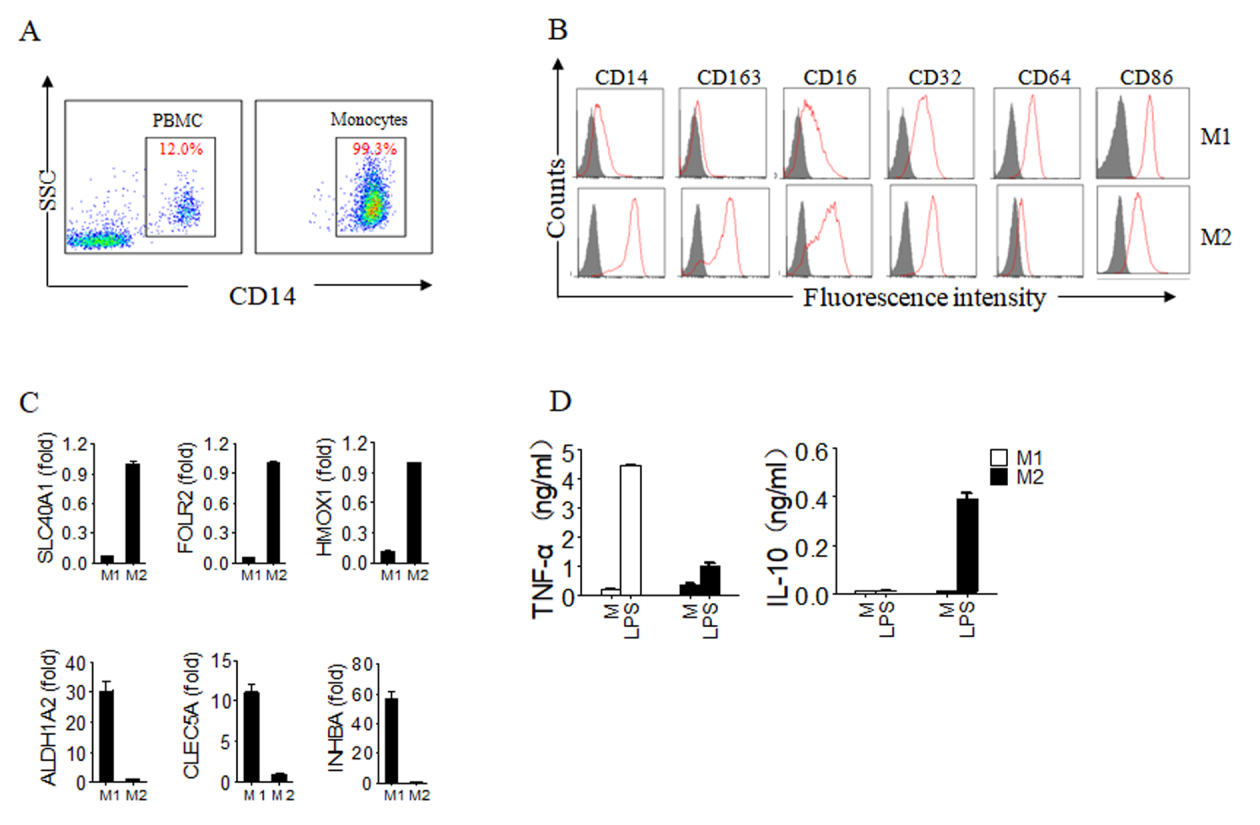


**Supplementary Figure 1.** (**GM-CSF)-M1, (M-CSF)-M2 macrophages differentiation and identification.** Monocytes were harvested by magnetic cell separation from PBMC. Cells were stained with PE-conjugated anti-human CD14 before and after separation and subjected to analysis by flow cytometry **(A)**. Human monocytes and monocyte-derived (GM-CSF)-M1/(M-CSF)-M2 macrophages were stained with APC-conjugated anti-human CD14, APC-conjugated anti-human CD163, APC-conjugated anti-human CD86, APC-conjugated anti-human CD16, APC-conjugated anti-human CD32, and FITC-conjugated anti-human CD64 for 30 minutes, and subjected to analysis by flow cytometry **(B)**. (GM-CSF)-M1 or (M-CSF)-M2 macrophages were collected and analyzed for mRNA expression of M2-specific markers including SLC40A1, FOLR2 and HMOX1, and M1-specific markers including ALDH1A2, CLEC5A and INHBA (normalized to GAPDH expression, fold change compared with M2) that was determined by quantitative RT-PCR **(C)**. Freshly differentiated (GM-CSF)-M1 or (M-CSF)-M2 macrophages (5×10^4^ cells/well) were incubated with 100 ng/ml LPS for 16 hours and the supernatants were collected. Concentration of TNFα and IL-10 was detected by using human TNFα and IL-10 ELISA kit **(D)**. The results are representative of three experiments from different donors.

**
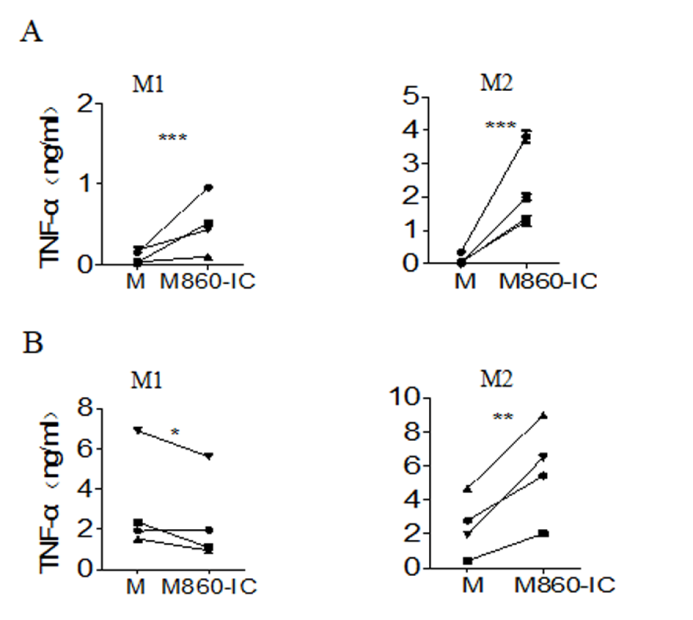
**

**Supplementary Figure 2. The effects of LTF-IC on M1 and M2 macrophages.** (GM-CSF)-M1 or (M-CSF)-M2 macrophages derived from different donors were stimulated with or without 30μg/ml M860-IC **(A)**, or re-stimulated by LPS after LTF-IC treatment **(B)**, TNFα were determined by ELISA kit. The results are representative of four experiments with different donors.
